# Germination physiology of *Cochlospermum fraseri* (Bixaceae), a deciduous tree from Northern Australia with physical seed dormancy

**DOI:** 10.1093/conphys/coad057

**Published:** 2023-09-01

**Authors:** Michael Just, Shane Turner, Adam Cross, Wolfgang Lewandrowski, Simone Pedrini, Kingsley Dixon

**Affiliations:** School of Molecular and Life Sciences, Curtin University, Kent Street, Bentley, Western Australia 6102, Australia; School of Molecular and Life Sciences, Curtin University, Kent Street, Bentley, Western Australia 6102, Australia; School of Molecular and Life Sciences, Curtin University, Kent Street, Bentley, Western Australia 6102, Australia; Ecological Health Network, 1330 Beacon St, Suite 355a, Brookline, Massachusetts 02446, United States of America; Department of Biodiversity, Conservation and Attractions, Kings Park Science, Fraser Avenue, Kings Park, Western Australia 6005, Australia; School of Biological Sciences, University of Western Australia, Stirling Highway, Crawley, Western Australia 6009, Australia; School of Molecular and Life Sciences, Curtin University, Kent Street, Bentley, Western Australia 6102, Australia; School of Molecular and Life Sciences, Curtin University, Kent Street, Bentley, Western Australia 6102, Australia

**Keywords:** Dormancy, Northern Australia, physiology, restoration, thermal performance

## Abstract

*Cochlospermum fraseri* (‘Kapok’, Bixaceae) is a deciduous tree widely distributed throughout semi-arid and monsoon tropical northern Australia, and an important species for ecological restoration in the region. We aimed to better understand the seed biology and ecology of *C. fraseri* to determine the mechanisms by which seed dormancy might be alleviated, and the conditions that support germination to inform the use of this species in restoration. Dormancy breaking treatments (wet heat, dry heat, scarification) commonly applied to species with physical seed dormancy (PY) were tested along with stratification at 5–35°C (nine treatments). Following dormancy alleviation, seeds were germinated at nine temperatures (5 to 40°C) and five water potentials (0 to −0.8 MPa) to understand environmental thresholds that regulate germination physiology in non-dormant seeds. A proportion of seeds (<0.3) lose dormancy naturally in response to warm (25 to 35°C) moist conditions, which dislodges the hypostase plug that prevents water uptake, whilst neither dry (≥100°C) nor wet (~100°C) heat were effective. Dormancy loss was also achieved by exposing seeds to concentrated (95–98% v/v) sulphuric acid for 3–7 hours, after which high proportions (>0.75) of germination were observed. *Cochlospermum fraseri* seeds possess PY, which is alleviated by seasonal temperatures that occur when soil moisture is high, allowing seeds to employ a risk-adverse strategy and maximize establishment success in episodic environments with stochastic rainfall events. The understanding of dormancy alleviation requirements gained here adds to our knowledge of PY worldwide and recruitment dynamics in the Australian monsoonal tropics and will aid land managers and restoration practitioners by informing both seeding sites and optimal time for in situ sowing as well as the potential capacity of this species to form a persistent soil seed bank.

## Teaser

Seeds possess complex mechanisms to ensure germination occurs when young vulnerable seedlings are most likely to survive. While crucial for species persistence, these mechanisms often pose a barrier in seed-based restoration. Through laboratory-based experiments we investigated these mechanisms in a species of tree from Northern Australia.

## Introduction

Seed germination is a critical life transition stage for plants, and the conditions that support seed germination and seedling recruitment are far more restrictive than those required for long-term vegetation survival (Hardegree *et al.,* 2018). Seeds of many species possess dormancy mechanisms, which time germination events to periods most conducive to seedling establishment (Baskin and Baskin, 2004). The timing of germination following dormancy alleviation is of particular importance in the context of ecological restoration, where seeds that have been treated to alleviate dormancy are then broadcast onto an area prepared for seeding (Kildisheva *et al.,* 2020; Shaw *et al.,* 2020; Turner *et al.,* 2022). Species able to cue dormancy release and germination to periods of above average rainfall are more likely to persist in environments with unpredictable hydroclimates (Long *et al.,* 2015). The significant periods of drought punctuated by intense rainfall events present in the Australian monsoonal tropics (AMT) significantly impact the timing of seeding efforts in restoration (Dalziell *et al.,* 2022). As well, the environmental conditions required for dormancy loss and germination for AMT species are largely unknown (Dalziell *et al.,* 2022). Understanding the impacts of water availability in combination with a full range of temperatures is imperative to facilitate successful restoration seeding and enhance biodiversity conservation in this largely understudied region.

The seasonality of precipitation in tropical regions is a major ecological driver of plant recruitment events, with increasing duration and intensity of the drought period and interannual rainfall variation strongly selecting for species reliant on the formation of persistent seed banks (Cross *et al.,* 2015a; Cross *et al.,* 2015b; Cross *et al.,* 2018). In the AMT, precipitation is dominated by tropical cyclones (TCs) and the Indo-Australian Summer Monsoon (IASM), which occur predominantly between December and March in association with the development of the IASM trough (Suppiah, 1992; Denniston *et al.,* 2015). Consequently, the region experiences substantial seasonal variability in summer rainfall, with monsoons and TCs capable of producing extreme volumes of rainfall in short periods of time, which coupled with high background evaporation, often result in extended dry periods between inundation events (Cross *et al.,* 2015a; Cross *et al.,* 2015b; Denniston *et al.,* 2015). As both the primary components of precipitation in the region, monsoons and TCs, are influenced by climate change (Denniston *et al*., 2015; Emanuel, 2013; Li *et al.,* 2013) it is critical that their importance in species recruitment dynamics is understood to better manage and restore ecosystems in the region.

The eastern Kimberly region of Western Australia harbours many plant families known to produce seeds with physical seed dormancy such as the Malvaceae, Fabaceae, and Sapindaceae (Western Australian Herbarium, 1998; Baskin and Baskin, 2014). Physical dormancy (PY) is imposed by water impermeable palisade cell layers in the outer seed or fruit coat and regulated by tightly sealed chalaza and micropyle openings termed the water gap, which become water permeable under specific soil conditions (Gama-Arachchige *et al.,* 2013). In the soil seedbank the opening of the water gap occurs in response to environmental signals, including moist or dry heat, and temperature fluctuations (Baskin and Baskin, 2014; Erickson *et al.,* 2016b). Seeds will germinate under the appropriate temperature and light conditions (Baskin and Baskin, 2014) once the water gap becomes unimpeded and sufficient moisture is available. Imbibition, which is typically rapid (Baskin and Baskin, 2014; Erickson *et al.,* 2016a; Gama-Arachchige *et al.,* 2012), occurs when these conditions are met.

While some plants adopt risk-taking strategies designed to rapidly germinate in response to small rainfall events over a wide range of temperatures, others adopt risk-averse strategies to only germinate in more hydrated soils over a narrow range of temperatures (Cross *et al.,* 2015b; Duncan *et al.,* 2019). Species may also lose dormancy, germinate and recruit variably in response to different rainfall scenarios, and in the future the success of these strategies may be influenced by climatic change (Ooi, 2012; Mark, 2015). Understanding the hydrothermal requirements for dormancy alleviation and germination is therefore crucial to maximize the probability of successful germination and establishment, thus increasing seed use efficiency in ecological restoration (Rajapakshe *et al.,* 2020; Just *et al.,* 2023).

*Cochlospermum fraseri* Planch. (‘Kapok’, Bixaceae) is a common deciduous tree widely distributed throughout semi-arid and monsoon tropical northern Australia (Atlas of living Australia, 2022), and an important species for ecological restoration in the region. The species was considered the only Western Australian representative of the genus prior to the recent discovery of the range-restricted shrub *Cochlospermum macnamarae* Hislop, K. R. Thiele & Brassington in the arid Pilbara bioregion (Hislop *et al.,* 2013). Worldwide, physical seed dormancy has been reported in the Bixaceae (Baskin and Baskin, 2014; Baskin and Baskin, 2021) and anecdotal evidence from Northern Australia suggests that scarified and untreated *C. fraseri* seeds broadcast into restoration sites emerge sporadically in low numbers, but the mechanisms by which dormancy might be overcome under natural conditions or by *ex situ* treatment remains undescribed.

We aimed to: better understand the seed biology and ecology of *C. fraseri* and determine the mechanisms by which seed dormancy might be alleviated to facilitate restoration seeding. Specifically, we aimed to (1) confirm that *C. fraseri* produces seeds possessing PY; (2) determine the optimum laboratory technique for alleviating dormancy and confirm the mechanism by which water enters the seed; and (3) investigate the hydrothermal conditions under which seeds lose dormancy and germinate. Results from this study have implications for the use of seeds under variable rainfall scenarios in restoration as well as the conservation of *Cochlospermum*, particularly the priority-listed (P4) range-restricted and poorly studied shrub *C. macnamarae* for which seeds have not yet been formally documented or described (Western Australian Herbarium, 1998).

## Methods

**Figure 1:**
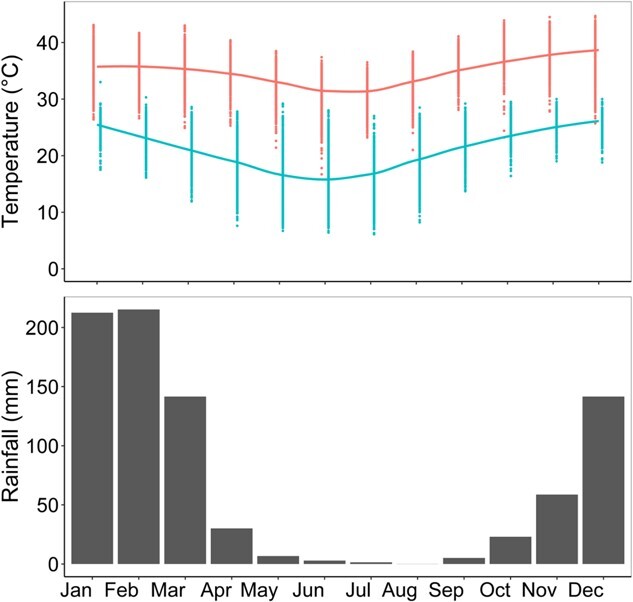
Daily maximum (top line) and minimum temperature (bottom line) and total average monthly rainfall recorded at the Bureau of Meteorology weather station 002056 (1971–2021) in the Eastern Kimberley.

The Eastern Kimberley experiences highly seasonal rainfall, with precipitation occurring almost entirely between December and March. The annual minimum and maximum mean daily temperatures range between 14.8°C and 39.1°C during the dry season, and between 24.9°C and 36.1°C during the wet season (Fig. 1) (Bureau of Meteorology, 2022).

### Seed origin, quality, morphology and dormancy assessment

Freshly dehiscent seeds of *C. fraseri* were collected from mature fruits in the Victoria Bonaparte bioregion of the East Kimberley (−16.71193, 128.436793) by Gelganyem Limited in September 2020 and stored under controlled conditions (ca. 21°C and 20% relative humidity) for up to 3 months prior to experimental use.

Seeds were inspected under X-ray (Autofocus X-ray cabinet, Faxitron, Tucson, USA) and scored as filled if the endosperm was fully developed and showed no signs of internal damage. Seed weight (mg) was determined from four replicates of 25 filled seeds, and the mean used to determine the thousand seed weight (TSW). The overall thickness of the testa and the presence and extent of palisade layer/s were determined by cutting five seeds in cross section and examining them under a scanning electron microscope (Jeol JCM 6000, Coherent Scientific, South Australia). Seeds to be imaged were first mounted onto 12.5 mm diameter aluminium pin stubs with pre-mounted carbon tabs. Pin stubs and seeds were then coated with gold for 30 seconds using a sputter coater (JEOL Smart Coater, Massachusetts, USA). Images were captured under high vacuum at 15 kV, and five measurements of testa thickness taken per seed before calculating the mean for each seed.

To assess if seeds are characterized as physically dormant, the rate of water uptake in untreated and nicked seeds was assessed from four replicates of five seeds per treatment by placing seeds on moistened Advantec 424, 84 mm filter paper (Toyo Roshi Kaisha, Ltd, Japan). For the seed nicking treatment, the testa of individual seeds was carefully scraped in a small, localized area under a binocular microscope using forceps and a scalpel to abrade the outer testa. An initial measurement of seed mass was conducted after five minutes, whereby seeds were blotted dry and weighed (time 0). Seeds were then placed back onto moist filter papers and reweighed after 1, 20, 24, 44, and 68 hours. The percentage water uptake was determined gravimetrically based on the fresh weight of non-imbibed seeds, with the percentage increase in seed mass calculated as:

$$ Water\ uptake=\frac{W_1-{W}_d}{W_{\mathrm{d}}}(100) $$

where *W_1_* and *W_d_* are the mean mass of imbibed and dry seeds, respectively (*sensu*Turner *et al.,* 2009).

**Figure 2:**
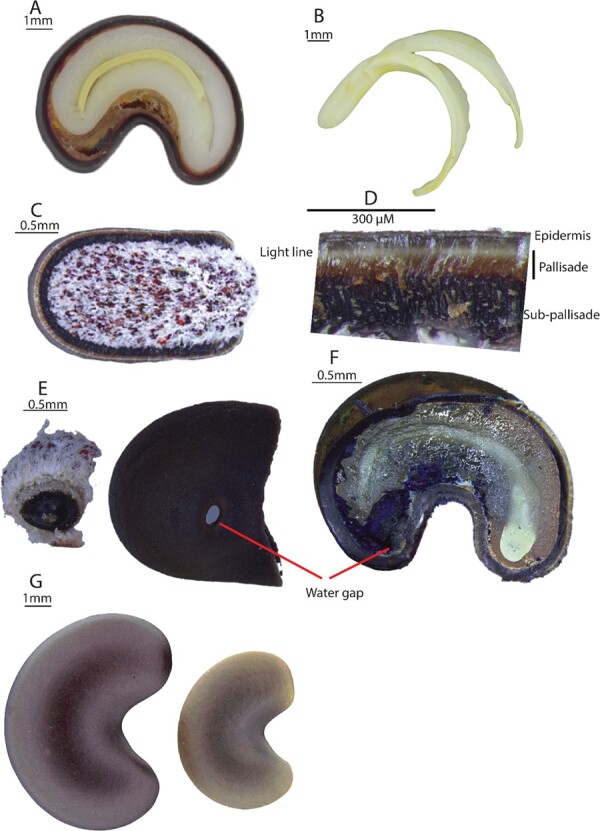
Select features of *C. fraseri* seeds. (A) Longitudinal section of a viable seed; (B) intact extracted embryo; (C) caudal view of seed with section of testa removed; (D) cross-section of testa showing the epidermis, light line, palisade and sub palisade layer; (E) hypostase plug; (F) location of water gap (left) and site of dye penetration of non-dormant seed (right); (G) non-dormant, imbibed seed (left) and dormant, non-imbibed seed (right).

To determine the site of water uptake in seeds, 10 untreated seeds and 10 seeds treated with 95–98% (v/v) H_2_SO_4_ for 3 hours, were soaked in a solution of methylene blue until seeds began to swell (~2 hours). Swollen seeds were then removed from the solution, patted dry and carefully dissected longitudinally. Cut sections were inspected under a light microscope (Leica M205C, Leica Camera, Wetzlar, Germany) and photographed at sites of dye infiltration (Figure 2F).

### Laboratory seed dormancy alleviation treatments

To determine whether scarification, hot water, dry heat, or acid exposure were effective in alleviating PY (as suggested in Kildisheva *et al.,* 2020). Scarification treatments were applied using a seed scarifier (Pneumatic Seed Scarifier PSS2000, Mater Seed Equipment, OEM Inc., Corvallis, OR, USA) fitted with 60, 120 or 180 grit sandpaper, with treatments including 10, 30, 60 or 120 seconds of scarification at 50 psi (Kildisheva *et al.,* 2018). Hot water treatments were applied by heating water to 100°C on a hot plate and immersing seeds for 1, 2, 4, 8 or 12 minutes, followed by immersion in room temperature (~23°C) water to allow seeds to rapidly cool. Dry heat was applied by dividing seeds into foil cups and placing the foil cups into a pre-heated oven at 100, 120, 140, 160, 180 or 200°C for 10, 30 or 60 seconds. For acid exposure, seeds were immersed in a 95–97%(v/v) H_2_SO_4_ solution for 0.5, 1, 2, 3, 4, 5, 6, 7 or 8 hours before transfer to an 8.4 g/L sodium bicarbonate solution for five minutes to neutralize the acid before seeds were rinsed three times in deionized water (Stevens *et al.,* 2015; Pedrini *et al.,* 2019). Following all treatments seeds were placed onto 0.7% (w/v) water agar in 90 mm Petri dishes and incubated at 25°C under a 12/12 h light/dark cycle in an incubator for four weeks. All treatments described were compared with untreated seeds that were subjected to the same incubation conditions and monitoring regime. Four replicates of 25 seeds were exposed to a representative range of each treatment. Seeds were scored for germination every 1 to 7 days and removed when the radical had extended to greater than 2 mm in length.

To determine if seeds of *C. fraseri* lose dormancy gradually over time in response to specific moist temperature conditions, fresh, untreated seeds were incubated on moistened Advantec 424, 84 mm filter paper (Toyo Roshi Kaisha, Ltd, Japan) in 90 mm Petri dishes at 5, 10, 15, 20, 25, 30 and 35°C. Another two sets of seeds were also incubated at 5°C or 35°C and moved either up (e.g. 5°C to 10°C) or down (e.g. 35°C to 30°C) respectively in 5°C increments every 4 weeks for 28 weeks, before reversing the direction of temperature change for another 28 weeks. These experiments are referred to as (1) 5 to 35°C and (2) 35°C to 5 throughout. Four replicates of 20 seeds were used for each of the nine temperature regimes tested. Seeds that imbibed water were found to increase in size substantially (Figure 2G), were soft when gently squeezed with forceps and thus considered to be non-dormant. Seeds were inspected weekly for 52 weeks and seeds that had increased in size were scored as non-dormant and removed. Seeds that became swollen within the first week were determined to be non-dormant at ‘time 0’ and were subtracted from the total number of seeds used in the statistical analysis (see below).

### Hydrothermal requirements of germination

To determine the hydrothermal optima for germination, dormancy was alleviated by exposing seeds to concentrated H_2_SO_4_ for three hours as previously described. After treatment, seeds were then surface sterilized in a 4% (w/v) sodium hypochlorite (NaOCI) solution supplemented with several drops of Tween 80 for 30 min under alternating vacuum (−70 kPA). Factorial combinations of incubation temperature and water stress were investigated by placing seeds onto Advantec 424, 84 mm filter paper (Toyo Roshi Kaisha, Ltd, Japan) in 90 mm Petri dishes containing 9 mm of polyethylene glycol (PEG) solutions calibrated to water stress levels of 0, −0.2, −0.4 and − 0.8 MPa (Michel, 1983). The Petri dishes were then carefully sealed with cling wrap to prevent moisture loss from evaporation during incubation. Four replicate Petri dishes per PEG solution were then incubated at 10, 15, 20, 25, 30, 35, 37 and 40°C for 30 days. Seed germination was scored every 2 days for 30 days, with germination defined as described previously.

### Statistical analysis

All analyses were conducted in the R statistical environment (R Core Team, 2022). Binomial generalized linear modelling (GLM), fitted with a logit-link function was employed to assess the effect of treatments (acid, hot water, or pneumatic scarification) on germination success or oven temperature on dormancy alleviation. The full model with interactions was fitted for each treatment and analysed to determine the main effects of treatment on germination success following four weeks incubation at the final temperature.

### Hydrothermal germination modelling

To understand the hydrothermal requirements to support germination, germination success over time for each temperature (10, 15, 20, 25, 30, 35, 37 and 40°C) was assessed using curvilinear log-logistic germination models (Ritz *et al.,* 2013). The drc package (Ritz *et al.,* 2015) was used to fit a three-parameter log-logistic function to germination data.

Eq 1 \begin{align*} germination=\frac{Gmax}{1+{\left(\frac{time}{\mathrm{t}50}\right)}^b} \end{align*}

Where *Gmax* is the upper limit for germination with the lower limit for germination rate assumed to be 0, *t*50 is the time required for germination to reach 50% from *Gmax*, and *b* is the slope of the germination function at *t*50. A full model was created for the number of germinated seeds over the number of seeds incubated for all temperature and stratification regimes. The *anova* function was used to assess the explanatory power of incubation duration and germination temperature and water stress as factors influencing *t*50 and *Gmax*, versus a global model without stratification duration, or germination temperature. Due to poor germination proportion following incubation at temperatures below 30°C (<0.25), 20 and 25°C incubation regimes were removed from the analysis.

Model fits for final germination proportion for hydrothermal requirements data were estimated using Beta, Yan and Hunt and Broken-Stick thermal performance functions described by Yin *et al.* (1995), Yan and Hunt (1999) and Yeager and Ultsch (1989) respectively. The AIC function was used to assess the explanatory power of each model and final model selection chosen accordingly (Supplementary Table 1). The final model was that described by Yin *et al.* (1995).

Eq 2 \begin{align*} &F(Temp)\notag\\&= Gmax{\left\{\left(\frac{Temp- Tbase}{Topt- Tbase}\right){\left(\frac{Tmax- Temp}{Tmax- Topt}\right)}^{\frac{Tmax- Topt}{Tmax- Topt}}\right\}}^b \end{align*}

where *Gmax* is the maximum germination proportion at any temperature (*Temp*), *Topt* is the optimum temperature for germination at the peak of the performance function, *Tmax* is the upper limit of thermal tolerance, *Tbase* is the lower limit of thermal tolerance and *b* is the slope of the function. This function was then fitted against the different water potential treatments, to determine how thermal performance was impacted by water stress.

We quantified the maximum germination response for each temperature to varying water potential and modelled the water potential threshold that limits germination by 50% (Ψb50) (Lewandrowski *et al.*, 2017) using the ED() function. We employed a variation of the three-parameter loglogistic equation, by fitting final germination responses against water stress.

Eq 3 \begin{align*} F(\boldsymbol{\Psi})=\frac{Gmax}{1+{\left(\frac{time}{\Psi \mathrm{b}50}\right)}^b} \end{align*}

the maximum germination proportion (Gmax) was the upper limit on the curve, b proportional to the slope of curve function F(Ψ), and the parameter Ψb50, the median response or inception point of the curve.

To quantify germination niche breadth, the thermal performance equation (Eq 2) was multiplied with the water stress performance equation (Eq 3) and the product of the two equations was plotted in a surface response heat map following the method of Dalziell *et al.,* 2022.

## Results

### Seed characteristics

**Figure 3:**
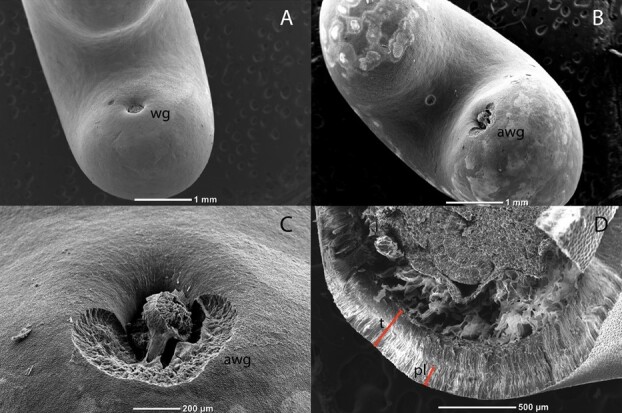
Scanning electron micrographs of *C. fraseri* seeds. (A) Fresh seed; (B) acid-treated seed; (C) water gap of acid-treated seed; (D) cross-section of fresh seed. Annotations are as follows: wg, water gap; awg, acid-treated water gap; t, testa; and pl, palisade layer.

The thousand seed weight of collected *C. fraseri* seeds was 47 ± 0.2 g. Seeds possessed a linear, fully developed embryo at the time of collection (Figure 2A-B). Freshly collected and untreated seeds exhibited a mass increase of 19 ± 3.16% following exposed to water for up to 68 hours, which is attributed to the imbibition of a small proportion (0.1) of seeds that were visibly swollen after this time (Figure 2G). In comparison, a significant increase in mass of 115 ± 0.54% was observed after 68 hrs for the nicked seeds following exposure to water, with 0.7 of seeds observed to have visibly swollen by this time (Figure 2G). The testa of fresh untreated seeds was 342 ± 8 μm thick, 68 ± 3 μm (~20%) of which was comprised of the palisade layer that is capped with several layers of outer epidermal cells 30 to 50 μM in thickness. A layer of sclerenchyma cells forms a subpalisade layer sitting beneath the palisade cells and is ~ 180 to 240μM thick (Figure 3D). Observation of non-dormant seeds (a proportion non-dormant at dispersal and those that had been acid treated) exposed to methylene blue showed the uptake of dye through the water gap (Figure 2F).

**Figure 4:**
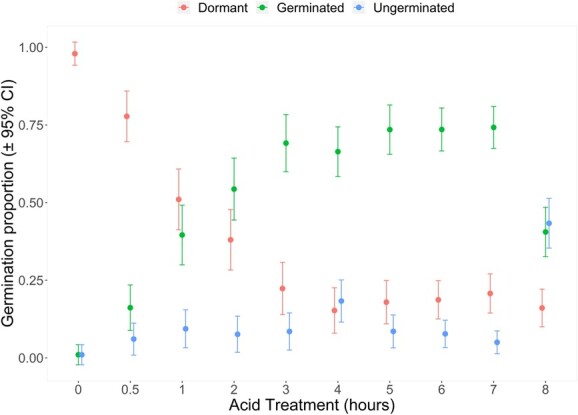
Proportion of dormant, germinated and ungerminated seeds incubated at 25°C following 0–8 hours in 95–97% H_2_SO_4_. Dormant seeds were those that failed to imbibe water, ungerminated seeds are those that imbibed water but failed to germinate after 4 weeks incubation.

Seeds subjected to including hot water, dry heat, or scarification treatments, showed extremely low germination rates (less than 0.07) regardless of the specific treatment combination used (Supplementary Table 2). Although dormancy loss was observed, indicated by seed swelling and softening, this was up to a maximum of 0.88 in seeds. This was especially prevalent with more extreme treatments, such as dry heat exposure of 140 to 200°C and prolonged hot water exposure of 8 and 12 minutes. Treatment with sulphuric acid increased germination significantly ranging from 0.16 to 0.74 (X^2^ = 308.81, d.f. = 9, *P* = 0.001), with acid treatment longer than 7 hours causing germination to decline (Figure 4). Optimal germination (0.7 to 0.75) was observed after exposure to acid for 3 to 7 hours with 0.15 to 0.2 of these seeds retaining dormancy and showing no signs of imbibition.

Incubation temperature had a significant effect on dormancy loss (X^2^ = 41.48, d.f. = 8, *P* = 0.001) (Supplementary Table 3). A low proportion of seeds lost dormancy at temperatures below 20°C (0.02–0.1), and seeds incubated between 25 and 35°C were more likely to lose dormancy after 52 weeks. When seeds were exposed to sequential temperatures, the proportion of dormancy lost at temperatures below 25°C was significantly less than at temperatures above 25°C.

### Seed germination modelling

**Figure 5:**
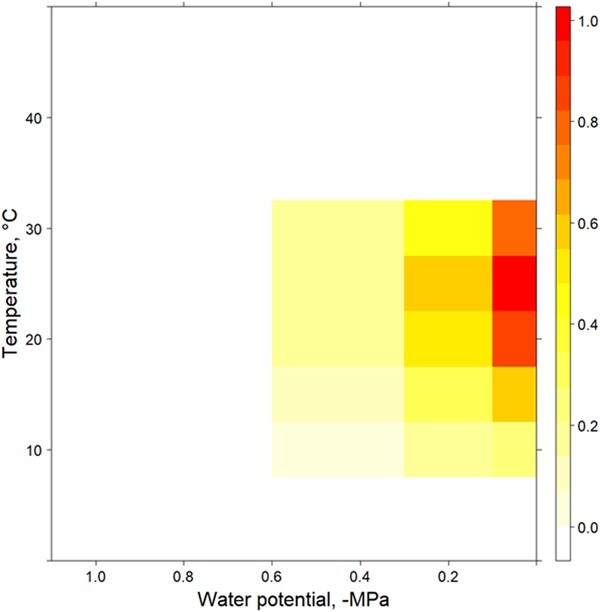
Hydrothermal germination niche for non-dormant *C. fraseri* seeds incubated between 10°C and 40°C at increasing water stress for 30 days. Different colours on scale shows model predictions of germination proportion at combinations of temperature (°C) and water potential (-MPa) once physical seed dormancy has been lost.

Germination proportion decreased from 1.00 to 0.58 as water stress increased from 0 to −0.4 MPa, and no germination was recorded at any temperature at −0.8 MPa (Table 1, Figure 5, Supplementary Figure 1). No significant difference was detected among optimum germination temperatures (*P* > 0.62, *t-value* = 34.63), or base temperatures (*P* > 0.9, *t-value* = 7.88) required for germination at increasing water stress (Table 1). The ceiling temperature at which germination becomes 0 was seen to decrease as water stress increased, with the difference between 0 MPa and − 0.4 MPa being significant (*P* < 0.0001, *t-value* = 14.67). As water stress increased, from 0 to −0.2 MPa (*P* < 0.0001, *t-value* = −7.18) and from −0.2 to −0.4 MPa (*P* = 0.009, *t-value* = −2.62), the time to attain 50% germination (*t*50) took significantly longer to achieve (Table 1). Across temperatures, the mean water potential at which 50% of seeds fail to germinate (Ψb50) was −0.38 ± 0.01 MPa. Seeds at 15–25°C had slightly lower Ψb50 (Supplementary Table 4) than seeds at 30°C, however there was only significant difference between 20 (−0.4 ± 0.01 MPa) and 30°C (−0.34 ± 0.01 Mpa) (*P* = 0.01, *t-value* = 2.61).

**Table 1:** Model estimates of *Gmax* following dormancy alleviation with sulphuric acid, *t*50 (days until 50% germination at 25°C) and thermal thresholds (°C) for germination of *C. fraseri* seeds incubated at 0, 15, 20, 25, 30, 35, 37 and 40°C under increasing water stress (0 to −0.8 MPa) for 30 days

	Water stress (MPa)			
Parameter	0	−0.2	−0.4	−0.8
*Gmax*	1.00 (±0.03)	0.96 (±0.04)	0.58 (±0.04)	0
*t*50	3.64 (±0.14)	4.66 (±0.15)	8.71 (±0.83)	NA
Base temperature	12.73 (±1.96)	12.76 (±2.12)	12.98 (±2.31)	NA
Optimum temperature	25.23 (±0.83)	25.72 (±2.05)	25.89 (±1.09)	NA
Ceiling temperature	37.71 (±0.43)	34.96 (±0.35)	30.11 (±0.22)	NA

## Discussion

*Cochlospermum fraseri* produces large seeds that disperse throughout most of the year in the AMT including the eastern Kimberley (Atlas of Living Australia, 2022). On-demand dormancy alleviation of *C. fraseri* has not been possible to date, leading to its absence from restoration sites across its natural range such as the former Argyle Diamond Mine in the East Kimberly. Anecdotal evidence suggests that sporadic germination occurs in the years following the direct return of seeds, but that uniform germination expected of seeds scarified to remove dormancy does not occur. As seen in the wider Bixaceae and other members of *Cochlospermum* (Baskin *et al.,* 2000; Harder *et al.,* 2007; Baskin and Baskin, 2014), this study has confirmed that the seeds of *C. fraseri* possess physical seed dormancy (PY).

### Seed dormancy alleviation treatments

We demonstrate that the conventional treatments to alleviate PY such as hot water, dry heat treatment or mechanical scarification are ineffective for this species. Freshly collected seeds possess a water gap, a fully developed campylotropous embryo, and largely fail to imbibe water without treatment to render the seeds water permeable. The lack of germination following scarification, dry heat and hot water treatments is likely due to the hardness of the seed and the thick testa present in *C. fraseri* (342 ± 8 μm) and the requirement for dislodgment of the bixoid chalazal plug to open the water gap, which proved to be completely resistant to conventional PY breaking treatments (Nandi, 1998; Baskin *et al.,* 2000). More aggressive treatment with mechanical scarification is likely to overcome dormancy but requires development and optimization either through extending the treatment time or the use of more abrasive grits (Kildisheva *et al.,* 2018). As well, both wet and dry heat treatments above 100°C are unlikely to break dormancy without the killing seeds in the process. As found with the co-occurring Kimberley species *Adansonia gregorii* (Australian boab), which also has tough seed coats and responds poorly to heat treatment (Turner *et al.,* 2009), this present study accomplished effective dormancy loss by exposing seeds to 98% sulphuric acid for 3 to 7 hours. This technique provides a simple, cost-effective and scalable method for dormancy alleviation in *C. fraseri* seeds with over 80% of seeds germinating in response to this treatment. However, as useful as it is, it provides no insight into the natural mechanism of dormancy alleviation for *C. fraseri* in the soil seed bank, nor it explains the observations of sporadic germination *in situ* that ecologists have noted years after restoration works have been completed.

### Hydrothermal requirements of dormancy loss and germination

While a range of high temperature treatments were found to be ineffective for promoting dormancy loss in the seeds of *C. fraseri*, we show that under the right soil conditions (warm moist soil conditions) a proportion of seeds will progressively lose dormancy over time with up to 30% of seeds becoming non dormant over 28 weeks when incubated at 35°C. Dormant seeds exposed to a range of temperatures under moist conditions lost dormancy more frequently at higher temperatures (25 to 35°C). This was particularly prominent for the two move-along treatments (5 to 35 to 5°C or 35 to 5 to 35°C) with seeds moved to warmer conditions (>25°C) losing dormancy more frequently, while those moved to cooler conditions (<25°C) showing fewer seeds losing dormancy. Interestingly, the temperatures required for dormancy alleviation by warm moist incubation (≥25°C to ≤ 35°C) neatly align with the optimum temperatures for germination as well as the *in situ* soil conditions recorded in parts of the Kimberley following rainfall events (Cross *et al.,* 2018). This indicates that dormancy alleviation occurs when soils are warm and moist, and consequently conditions are conducive to both seed germination and seedling establishment. These results provide empirical evidence for field observations of *C. fraseri* emerging stochastically in restoration sites months or even years after seeding, and add to the number of species with PY reported to lose dormancy under warm (25°C to 35°C) moist conditions rather than high (>50°C) temperatures (Cook *et al.,* 2008; Turner and Dixon, 2009). While seasonal dry periods may contribute to dislodging the water gap in the seeds of some physically dormant species (Gama-Arachchige *et al.,* 2012), the evidence gathered here suggests that dormancy alleviation of *C. fraseri* is intimately tied to water availability.

### Seed ecology of *C. fraseri*

When water is non-limiting seed germination is fast and non-dormant seeds germinate over wide thermal range. However, as water stress increases, both the proportion of seeds germinating and the thermal range over which germination occurs significantly decreases. Together with the relatively rapid germination speed these results suggest that *C. fraseri* relies on larger rainfall events, and that moisture needs to be retained for up to 8 days under relatively wet conditions. Germination, however, could also be completely opportunistic given the wide thermal envelope. In seasons with suboptimal soil moisture, we believe in the context of our results that seedling recruitment of *C. fraseri* is likely to be highly restrictive, as seeds are precluded from both dormancy loss and germination by environmental conditions that do not overlap with their core requirements. Modelling the germination niche across gradients of relevant environmental variables can inform both restoration and conservation activities. For example, the models in this study could aid in the development of predictive models of species occurrence, like the Terrain Predictive Model developed to survey the range restricted *Conospermum toddii* in the remote Great Victoria Desert (Murdock *et al.,* 2010), an approach that could be applied to *C. macnamarae,* which has a highly localized distribution and is currently of some conservation concern (P4) (Hislop *et al.,* 2013). It is important to note, however, that the models presented here are theoretical estimates of the germination niche and tend to overestimate germination proportion at the hydrothermal limits (pale yellow to white on scale). The techniques required to understand seed ecology and population distributions also enable the effective use of species in restoration by informing best practice seed pre-treatments, the timing of direct seeding, and the production of tubestock (Pedrini and Dixon, 2020; Turner *et al.,* 2022). While on-demand germination treatments are a key component of restoration seeding, it is important to consider natural mechanisms, particularly when dealing with stochastic environmental conditions, short seeding windows, and complex seed ecology.

The proportion of dormancy loss by warm moist incubation observed here suggests that cohorts of *C. fraseri* seeds possess a spectrum of PY alleviation thresholds, the majority of which are not alleviated in the first wet season following dispersal. Many species are known to have limited seed germination in the first season following dispersal (Just *et al.,* 2019; Dalziell *et al.,* 2022; Turner *et al.,* 2022). Seed dormancy which limits germination in a season is common in temporally variable environments (Baskin and Baskin, 2003), so this is not an unexpected result for the seeds of *C. fraseri*. The low rate of dormancy loss, the warm wet conditions required to alleviate dormancy, and the overlap between dormancy alleviation and germination requirements suggest that recruitment of *C. fraseri* seedlings is mediated by multiple processes displaying a ‘risk-avoidance’ strategy, limiting dormancy loss and germination to periods of elevated soil moisture during the hot summer months, and spreading germination events across multiple seasons to minimize seedling mortality in years with low rainfall (Duncan *et al.,* 2019).

Understanding the hydrothermal requirements for both dormancy alleviation and germination in PY species is critical for restoration in extreme episodic environments. This knowledge can be used to develop nuanced seeding strategies that capitalize on natural dormancy mechanisms, limit risk under uncertain rainfall scenarios and better manage restored environments knowing that some species are likely to persist in the soil seed bank for much longer than others so require less focus to return. This could help inform supplementary irrigation, as well as manipulations to microsites to increase soil water retention. Future research should further adapt the approaches developed here and apply them to a wider range of species required for restoration, focusing on the hydrothermal requirements of seeds for germination and the conditions needed to promote dormancy alleviation in soil. Understanding the seed ecology of these species over multiple seasons will help to develop models of dormancy loss and the germination niche, and significantly benefit seeding activities by informing optimum seeding sites and times.

## Conclusions

Here we provide evidence for PY in one of the two Bixaceae species occurring in Australia and show that dormancy is naturally alleviated by warm moist conditions rather than high temperatures as is so commonly found in many other species with PY. Ecologically, recruitment events for *C. fraseri* are possible during any time of the year given higher water availabilities. However, as seen in other arid zone species, significant recruitment pulses of this species is likely rare and relies upon significant rainfall events. The understanding of dormancy alleviation requirements gained here, coupled with the hydrothermal germination models, adds to our understanding of PY and recruitment dynamics in the AMT zone and will aid land managers and restoration practitioners in areas containing *Cochlospermum* species. Restoration practitioners may also benefit from this knowledge as we now know the critical temporal and climactic thresholds driving germination.

## Supplementary Material

Web_Material_coad057
