# Supplementary material for: Germination physiology of Cochlospermum fraseri (Bixaceae), a deciduous tree from Northern Australia with physical seed dormancy
Source: Conserv Physiol. 2023 Sep 1;11(1):coad057. doi: 10.1093/conphys/coad057 (PMC10476509; doi:10.1093/conphys/coad057)
Supplement: Web_Material_coad057 [file web_material_coad057.docx]

**Supplementary**

Supplementary Table 1. AIC values used to select the best fit model for hydrothermal germination response of *C. fraseri*.

| Model | Degrees of freedom | AIC |
| --- | --- | --- |
| Yin, Kropff, Mclaren and Visperas (1995) | 16 | -244.3243 |
| Yeager and Ultsch (1989) | 16 | -244.1818 |
| Yan and Hunt (1999) | 10 | -160.3868 |

Supplementary Table 2. Maximum germination achieved within dormancy breaking experiments (n=100)

| Treatment | Maximum germination  (%) |
| --- | --- |
| Hot water (98 °C for 1 to 12 mins) | 0.06 (±0.05) |
| Dry Heat (10 to 60 s exposure to 100, 120, 140, 160, 180 or 200 °C) | 0.02 (±0.02) |
| Pneumatic Scarification (10 to 120 s exposure to 60, 120 or 180 grit) | 0.06 (±0.05) |


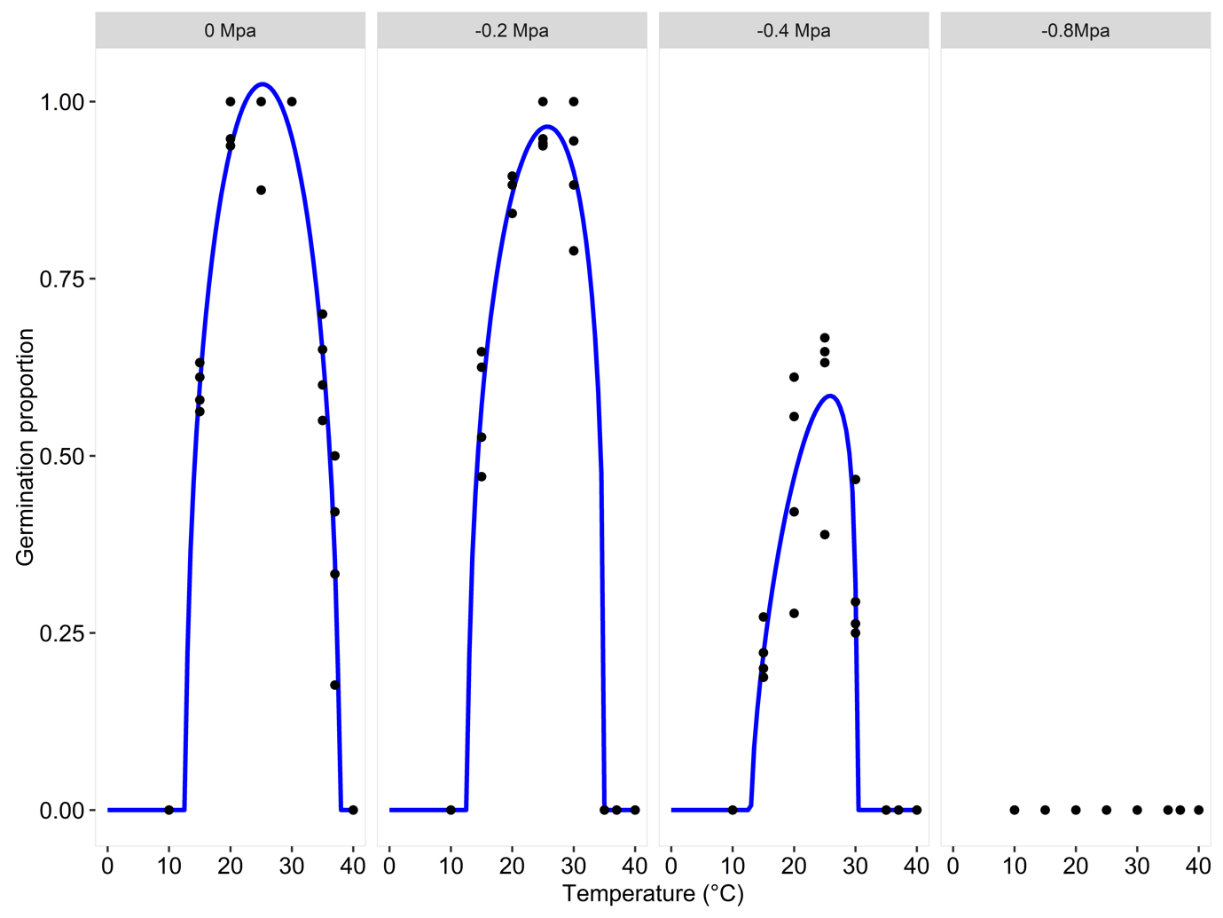
Supplementary Figure 1. Hydrothermal germination models for *Cochlospermum fraseri* seeds incubated between 10 – 40°C at increasing water stress for 30 days

Supplementary Table 3**.** Proportion of fresh *Cochlospermum fraseri* seeds (± CI) that had lost dormancy (*n* = 80) following incubation at either one of seven constant temperatures (5-35°C) for 52 weeks or monthly movement from 5 to 35°C or from 35 to 5°C in 5°C increments every 4 weeks. Seeds were checked weekly and seeds that had either germinated or had lost dormancy (i.e., were swollen and soft – Figure 2G) were counted. Grey shading (28 wks) indicates when both move long treatments (5°C to 35°C to 5°C or 35°C to 5°C to 35°C) had reached the halfway mark (i.e. 35°C or 5°C).

|  | Weeks of incubation | | | | | | | | | | | | |
| --- | --- | --- | --- | --- | --- | --- | --- | --- | --- | --- | --- | --- | --- |
| Temperature  (°C) | 4 | 8 | 12 | 16 | 20 | 24 | 28 | 32 | 36 | 40 | 44 | 48 | 52 |
| 5 to 35 to 5 | 0.01  (0.00 to 0.07) | 0.03  (0.00 to 0.09) | 0.04  (0.01 to 0.11) | 0.06  (0.02 to 0.14) | 0.08  (0.03 to 0.16) | 0.10  (0.05 to 0.19) | 0.16  (0.10 to 0.26) | 0.16  (0.10 to 0.26) | 0.19  (0.12 to 0.29) | 0.21  (0.14 to 0.32) | 0.24  (0.16 to 0.34) | 0.24  (0.16 to 0.34) | 0.24  (0.16 to 0.34) |
| 5 | 0.04  (0.01 to 0.11) | 0.04  (0.01 to 0.11) | 0.04  (0.01 to 0.11) | 0.08  (0.03 to 0.16) | 0.08  (0.03 to 0.16) | 0.08  (0.03 to 0.16) | 0.08  (0.03 to 0.16) | 0.08  (0.03 to 0.16) | 0.09  (0.04 to 0.17) | 0.11  (0.06 to 0.20) | 0.11  (0.06 to 0.20) | 0.11  (0.06 to 0.20) | 0.11  (0.06 to 0.20) |
| 10 | 0.01  (0.00 to 0.07) | 0.01  (0.00 to 0.07) | 0.01  (0.00 to 0.07) | 0.03  (0.00 to 0.09) | 0.03  (0.00 to 0.09) | 0.03  (0.00 to 0.09) | 0.03  (0.00 to 0.09) | 0.03  (0.00 to 0.09) | 0.03  (0.00 to 0.09) | 0.03  (0.00 to 0.09) | 0.03  (0.00 to 0.09) | 0.03  (0.00 to 0.09) | 0.03  (0.00 to 0.09) |
| 15 | 0.03  (0.00 to 0.09) | 0.04  (0.01 to 0.11) | 0.04  (0.01 to 0.11) | 0.05  (0.02 to 0.13) | 0.08  (0.03 to 0.16) | 0.08  (0.03 to 0.16) | 0.08  (0.03 to 0.16) | 0.08  (0.03 to 0.16) | 0.09  (0.04 to 0.17) | 0.09  (0.04 to 0.17) | 0.09  (0.04 to 0.17) | 0.09  (0.04 to 0.17) | 0.10  (0.05 to 0.19) |
| 20 | 0.05  (0.02 to 0.13) | 0.05  (0.02 to 0.13) | 0.05  (0.02 to 0.13) | 0.05  (0.02 to 0.13) | 0.05  (0.02 to 0.13) | 0.06  (0.02 to 0.14) | 0.06  (0.02 to 0.14) | 0.06  (0.02 to 0.14) | 0.06  (0.02 to 0.14) | 0.08  (0.03 to 0.16) | 0.08  (0.03 to 0.16) | 0.08  (0.03 to 0.16) | 0.10  (0.05 to 0.19) |
| 25 | 0.09  (0.04 to 0.17) | 0.09  (0.04 to 0.17) | 0.10  (0.05 to 0.19) | 0.10  (0.05 to 0.19) | 0.10  (0.05 to 0.19) | 0.10  (0.05 to 0.19) | 0.13  (0.07 to 0.22) | 0.14  (0.08 to 0.23) | 0.14  (0.08 to 0.23) | 0.15  (0.09 to 0.25) | 0.15  (0.09 to 0.25) | 0.18  (0.11 to 0.27) | 0.19  (0.12 to 0.29) |
| 30 | 0.05  (0.02 to 0.13) | 0.11  (0.06 to 0.20) | 0.13  (0.07 to 0.22) | 0.13  (0.07 to 0.22) | 0.14  (0.08 to 0.23) | 0.14  (0.08 to 0.23) | 0.15  (0.09 to 0.25) | 0.16  (0.10 to 0.26) | 0.18  (0.11 to 0.27) | 0.18  (0.11 to 0.27) | 0.19  (0.12 to 0.29) | 0.23  (0.15 to 0.33) | 0.23  (0.15 to 0.33) |
| 35 | 0.05  (0.02 to 0.13) | 0.09  (0.04 to 0.17) | 0.09  (0.04 to 0.17) | 0.15  (0.09 to 0.25) | 0.25  (0.17 to 0.36) | 0.26  (0.18 to 0.37) | 0.29  (0.20 to 0.40) | 0.33  (0.23 to 0.43) | 0.33  (0.23 to 0.43) | 0.34  (0.24 to 0.45) | 0.35  (0.25 to 0.46) | 0.35  (0.25 to 0.46) | 0.39  (0.29 to 0.50) |
| 35 to 5 to 35 | 0.13  (0.07 to 0.22) | 0.16  (0.10 to 0.26) | 0.21  (0.14 to 0.32) | 0.23  (0.15 to 0.33) | 0.24  (0.16 to 0.34) | 0.24  (0.16 to 0.34) | 0.24  (0.16 to 0.34) | 0.24  (0.16 to 0.34) | 0.24  (0.16 to 0.34) | 0.26  (0.18 to 0.37) | 0.26  (0.18 to 0.37) | 0.30  (0.21 to 0.41) | 0.31  (0.22 to 0.42) |

Supplementary Table 4. Model fits of Ψb50 calculated from three parameter loglogistic models

| Temperature (°C) | Ψb50 | Std. Error | t-value | p value |
| --- | --- | --- | --- | --- |
| 15 | -0.362422 | 0.026806 | 13.5204 | < 2.2e-16 |
| 20 | -0.397298 | 0.014104 | 28.1694 | < 2.2e-16 |
| 25 | -0.419004 | 0.040249 | 10.4104 | 2.54E-14 |
| 30 | -0.336868 | 0.015486 | 21.7536 | < 2.2e-16 |
